# MEMS and FOG Technologies for Tactical and Navigation Grade Inertial Sensors—Recent Improvements and Comparison

**DOI:** 10.3390/s17030567

**Published:** 2017-03-11

**Authors:** Olaf Deppe, Georg Dorner, Stefan König, Tim Martin, Sven Voigt, Steffen Zimmermann

**Affiliations:** Northrop Grumman LITEF GmbH, 79115 Freiburg, Germany; Deppe-Reibold.Olaf@ng-litef.de (O.D.); Dorner.Georg@ng-litef.de (G.D.); Koenig.Stefan@ng-litef.de (S.K.); Martin.Tim@ng-litef.de (T.M.); Voigt.Sven@ng-litef.de (S.V.)

**Keywords:** MEMS accelerometer, MEMS gyroscope, coriolis vibratory gyroscope, fiber optic gyroscope, multifunction integrated optics chip, angle random walk

## Abstract

In the following paper, we present an industry perspective of inertial sensors for navigation purposes driven by applications and customer needs. Microelectromechanical system (MEMS) inertial sensors have revolutionized consumer, automotive, and industrial applications and they have started to fulfill the high end tactical grade performance requirements of hybrid navigation systems on a series production scale. The Fiber Optic Gyroscope (FOG) technology, on the other hand, is further pushed into the near navigation grade performance region and beyond. Each technology has its special pros and cons making it more or less suitable for specific applications. In our overview paper, we present latest improvements at NG LITEF in tactical and navigation grade MEMS accelerometers, MEMS gyroscopes, and Fiber Optic Gyroscopes, based on our long-term experience in the field. We demonstrate how accelerometer performance has improved by switching from wet etching to deep reactive ion etching (DRIE) technology. For MEMS gyroscopes, we show that better than 1°/h series production devices are within reach, and for FOGs we present how limitations in noise performance were overcome by signal processing. The paper also intends a comparison of the different technologies, emphasizing suitability for different navigation applications, thus providing guidance to system engineers.

## 1. Introduction

As an industry provider of navigation systems, gyrocompasses and attitude and heading reference systems (AHRS), we start our discussion driven by industry practice and long term experience by introducing the basic inertial building blocks, then turn to specific inertial system level requirements driven by customers and, at the end of this section, introduce the inertial sensor technologies we provide for our series products based on several decades of experience in the field. For our most recent improvements we have dedicated sections to each type of inertial sensor.

The terminology of inertial systems can be summarized as follows [1,2]: An Inertial Sensor Assembly (ISA) is obtained through the combination of accelerometers and gyroscopes in defined orientations. An Inertial Measurement Unit (IMU) uses an ISA in order to measure the movement of its body in three-dimensional space without external reference. An inertial system uses an IMU and external aids could be employed, such as a magnetic compass, airspeed data, barometric altitude, star tracker, satellite navigation systems (GNSS), etc.

Some typical links between sensor performance and system functionality are: An Inertial Reference System (IRS) for aviation estimates an aircraft’s position, velocity, and attitude with respect to the earth. An IRS requires an ‘inertial navigation quality’ IMU with a gyroscope performance of 0.01°/h and better. IMUs with a gyroscope performance between 0.01°/h to 5°/h are used in attitude and heading reference systems (AHRSs). An AHRS primarily estimates the attitude angles of an aircraft. Gyroscopes with 0.1°/h and better enable the capability to perform coarse navigation and to gyrocompass. AHRS with 1°/h gyroscopes need external references, such as magnetic compass, to find the north direction. Although the integration of IMU and Global Navigation Satellite Systems (GNSS) has a lot of advantages, a benefit for air navigation can be generated with a gyro performance of 0.1°/h or better. More about the role of inertial systems in the environment of GNSS is described in [3].

Inertial systems in GNSS-denied environments and areas where GNSS are simply not available are receiving more and more attention again. For example, the main accuracy requirement of the NG LITEF Drill Pilot used in drilling and mining is defined in terms of the position error relative to the respective bore distance, i.e., position error (1σ) ≤ 0.5%. In addition, the continuous rotation around the longitudinal axis with a high angular rate (up to 700°/s) during drilling operations represents a major challenge with respect to the sensors and their processing [4].

For the design of inertial systems, sensor performance data and error models are crucial, and numerical system simulations are an appropriate tool to support the design and evaluation phases. Reliable simulation tools are more than helpful during the validation and verification process for complex integrated systems intended to be certified. A suitable tool chain, developed by NG LITEF, is presented in [5]. Manufacturers that have a deep understanding about the inertial sensors and their performance characteristics are in a good positon to design optimized inertial systems, namely the effectiveness in terms of performance and cost.

During the last decades, sensor types without moving parts such as Sagnac effect-based gyroscopes have replaced mechanical gyroscopes which require rather short maintenance intervals. For instance, the Fiber Optic Gyroscope (FOG) [6] allows for performance scaling through the fiber coil length and navigation grade performance has been reported for FOGs. However, mechanical inertial sensors have reentered the arena due to the progress in microelectronics and associated silicon processing in the form of MEMS devices.

MEMS-based sensors do not require maintenance of bearings, are suitable to be manufactured in mass production, and were first introduced for accelerometers, and a little later also for gyroscopes. Silicon is a very robust material with advantageous properties borrowed from metals, including low electrical resistivity, but with practically no wear-out when moved or bent within elastic region. On the other hand, silicon dioxide is an excellent insulator, easy to process and perfectly matches the mechanical and thermal properties of silicon. Layers of silicon separated by silicon dioxide are called Silicon-On-Insulator (SOI) wafers and can be purchased as raw material. Among technologies adopted for MEMS fabrication, two main Si-based branches have evolved: (i) Si-bulk technology with KOH wet etching and (ii) Deep Reactive Ion Etching (DRIE) and its various derivatives originating from the classic Bosch process [7]. The chip assembly relies on wafer bonding in both cases. While Si-bulk technology is still widely used for MEMS accelerometers, for MEMS gyroscopes—apart from a few early exceptions [8]—nowadays Deep Reactive Ion Etching is applied, owing to structural complexity of the devices [9].

Having introduced the available sensor technologies and the requirements flow-down from the system level to inertial sensor performance characteristics, we provide an overview of recent improvements in NG LITEF MEMS accelerometers in Section 2. We demonstrate that the introduction of the proof mass as a laterally moving structure along with an optimized DRIE bridge technology has greatly improved sensor performance at a higher efficiency. Latest developments in MEMS gyroscopes are addressed in Section 3, where we emphasize the suitability of an optimized dual-mass Lin-Lin-design in harsh environments including vibration and acoustic noise. NG LITEF’s Fiber Optic Gyroscopes have been optimized over more than two decades [10,11,12,13,14], nevertheless some parasitic effects of the Multifunction Integrated Optics Chip (MIOC) have so far limited the overall high end FOG performance. In Section 4, we present solutions to significantly reduce these limitations. We complement the discussion with a brief comparison of FOG and MEMS technologies and their suitability for different applications in Section 5, assuming that this adds value to the paper for readers with a system engineering background who are searching for selection criteria.

## 2. MEMS Accelerometers

### 2.1. Background

For micromechanical ‘pendulum-type’ accelerometers with capacitive readout and torqueing, designs with vertical (out-of-plane) or lateral (in-plane) displacements are commonly used. Throughout Section 2 these are referred to as vertical and lateral designs.

In the accelerometer products of NG LITEF, both design concepts are realized. The vertical design as used in [15] was developed in the 1990s, whereas the lateral design was introduced in the past few years. In the following, the two concepts are analyzed theoretically. The results of the analysis are then supported by production data of the two different accelerometer types. At the end of Section 2, a short outlook into the near future is given.

### 2.2. Theoretical Analysis

The capacity $C_{0}$ of a parallel plate capacitor is given by

(1) $$C_{0}\;=\;N\cdot \frac{\varepsilon_{0}\cdot \varepsilon_{R}\cdot A}{d}\;=\;N\cdot \frac{\varepsilon_{0}\cdot \varepsilon_{R}\cdot L\cdot h}{d}\;,$$

where L and h are dimensions of the plate and d is the gap between the two plates. N is the total number of electrodes.

#### 2.2.1. Electrode Design: Geometrical Sensitivity

**Figure 1:**
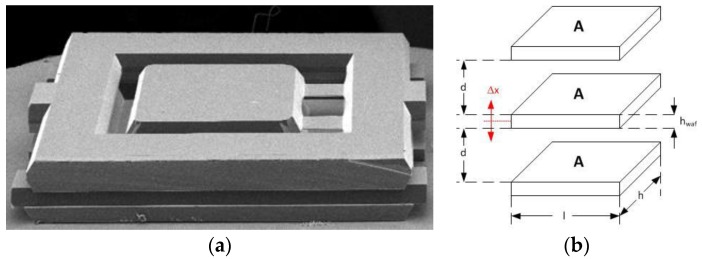
Accelerometer Vertical Design: (**a**) SEM image without top electrode; (**b**) Electrode scheme, including top electrode.

The geometrical sensitivity γ of an accelerometer to displacements x is given by the derivative of the capacity by displacement. In designs with vertical sensing, the sensitive direction (input axis x) of the single (N = 1) accelerometer electrode is in direction of d, as shown in Figure 1.

Hence the sensitivity of the vertical design is

(2) $$\gamma_{V}\;\approx \;\frac{\varepsilon_{0}\cdot \varepsilon_{R}\cdot A}{d^{2}{-x}^{2}}\;\approx \;\frac{\varepsilon_{0}\cdot \varepsilon_{R}\cdot A}{d^{2}}\;.$$

For vertical designs, wet etching processes can be used. Large pendulum sizes A of e.g., 1 × 1 mm^2^ can be structured within a wafer, whereas the gap d between the parallel plates is adjusted by removing specific silicon oxides from top and bottom electrodes. Thus, tiny gaps of the order of a few microns are easily attainable. Vertical designs, processed with KOH, were the first MEMS accelerometer designs, not only at NG LITEF. With the rough numbers given above, geometrical sensitivities of the order >10^−6^ F/m = 1 pF/µm can be realized.

With wet etching processes, high aspect ratios (the ratio of the size to the height of structures, such as gaps or trenches) are not achievable, since different crystal lattice orientations show largely different etching rates. Thus, wet-etched accelerometer designs most likely show features with angles of 54.74° as can also be seen in Figure 1.

Later, Deep Reactive Ion Etching (DRIE) was developed, in particular with the development of the Bosch process [7]. Although the 54.74° limitation of wet etching processes does not exist for DRIE, there is still a limit for the maximum aspect ratio that must be obeyed. The actual limit depends on the etching machines and the level of experience of the MEMS technologists operating them. In order to allow for gap sizes of a few microns, the active layer thickness is of the order of 100 µm maximum at present. For newer designs, DRIE processes are utilized to structure the wafer.

In vertical DRIE designs, the pendulum size is smaller. It is given by the product of the thickness of the active layer times the electrode length. This results in an electrode area of the order of 0.05 mm^2^ maximum. Thus, in vertical DRIE designs between 10 and 50 electrodes are required to produce a similar geometrical sensitivity as a vertical wet-etched design with a single pendulum electrode.

**Figure 2:**
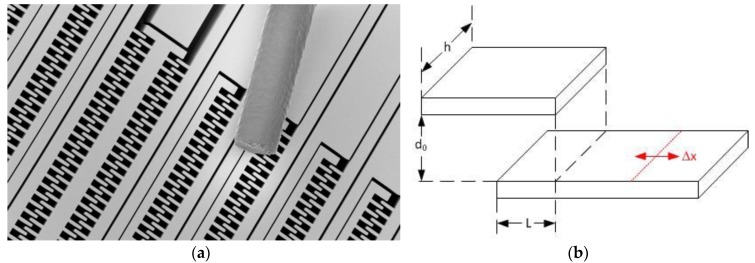
Accelerometer Lateral Design: (**a**) SEM image showing the large number of electrodes needed to achieve reasonable sensitivity; (**b**) Electrode scheme.

In lateral DRIE designs, the length L is changed through external accelerations by ±Δx. Here, the symbol h refers either to the thickness of the device wafer (the pendulum height), as in Figure 2.

In lateral designs, the sensitivity is

(3) $$\gamma_{L}\;=\;2\cdot N\cdot \frac{\varepsilon_{0}\cdot \varepsilon_{R}\cdot h}{d}\;.$$

The aspect ratio (h/d) puts a universal limit on the sensitivity of a single electrode. For an aspect ratio of h/d ≈ 18, one finds a geometric sensitivity of only 3 × 10^−10^ F/m = 0.3 fF/µm. Newer developments [16] utilize a gap reduction after DRIE. This method allows increasing the maximum aspect ratio by a factor of 5 to 10. Nevertheless, the relationship between the sensitivity of vertical to lateral designs is at least of the order of several hundreds to a thousand for a single electrode. Thus, the number of electrodes must be increased in lateral design in order to compensate the lack of sensitivity, as shown in Figure 2.

#### 2.2.2. Spring Design and Displacement vs. Acceleration

At low frequencies, the displacement x of the pendulum is proportional to the acceleration by

(4) $$x\;=\;\frac{a}{\omega_{0}^{2}}\;,$$

where $\omega_{0}$ is the undamped resonant frequency of the pendulum, determined by the ratio of the spring constant k and the mass m of the pendulum. In order to obtain a high sensitivity to acceleration, the resonant frequency must be low. This can be obtained with a spring design that provides a small spring constant in the sensitive direction, but has a high stiffness in the perpendicular directions (in order to suppress cross-axis sensitivities). Alternatively, the spring constant in the sensitive direction can be adjusted electrostatically [17].

The product of geometrical sensitivity and the displacement/acceleration relationship yields the capacitive sensitivity of the MEMS chip to accelerations. If a high bias stability of the accelerometer is desired, it is important to have a high capacitive sensitivity to accelerations, so that parasitic capacitive impacts are suppressed effectively. For designs with a lower acceleration sensitivity, as for example in lateral designs, special care must be taken in the packaging, MEMS die attachment, layout, and bonding of the sensitive lines to the front-end electronics.

#### 2.2.3. Forces: Linearity

The force F in a parallel plate capacitor with constant voltage U is

(5) $$F_{\max}\;=\;\gamma_{V/L}\cdot \frac{U^{2}}{2}\;.$$

For vertical designs, the force depends on the pendulum position (the sensitivity depends on the displacement x), whereas in lateral designs the maximum acceleration is independent from the pendulum position. This means that torque and pickoff signals do depend on the pendulum position in a non-linear way in vertical designs. Consequently, a linearization procedure has to be performed in accelerometers operating with vertical displacement if good performance is required. In contrast, accelerometers with lateral displacement do not require such additional linearization procedure.

When the feedback force is largely independent from the pendulum displacement, an excellent behavior of the accelerometer, even under high vibration levels, can be expected.

Since the force determines the accelerometer scale factor, a positive impact on its progression over temperature should be observable. This statement is true in particular for lateral designs, where a position independent force is reached by design.

A further impact on the accelerometer scale factor is given by the fact that in vertical designs processed with wet etching, the top and bottom electrodes are subject to the outside air pressure. Changes in the pressure can alter the electrode distances and thus the scale factor. This impact is particularly visible in non-hermetic accelerometer packages for obvious reason, in non-evacuated hermetic packages the impact can be seen as a progression with temperature. In lateral designs and in vertical design processed with DRIE, the electrode distance is normally not in a pressure sensitive direction.

#### 2.2.4. Damping, Noise, and Bandwidth

In vertical designs, squeeze film damping is dominant between the two parallel plates moving on each other. Even with a low pressure in the MEMS device, a high damping coefficient can be obtained. In contrast, the squeeze film effect does not play an important role in lateral designs, since the electrodes do not move over each other. Thus, special damping electrodes are required if large displacements shall be avoided. In addition, the gas pressure must be significantly higher in comparison to vertical designs. Unfortunately, this causes a higher thermomechanical noise from the Brownian motion of the fill gas.

Damping very often is a tradeoff between thermomechanical noise and vibration rectification error (VRE). The latter can be partly compensated even with a lower damping coefficient if the accelerometer is operated closed-loop and the bandwidth is sufficiently high. In NG LITEF’s current accelerometer products, the vibration rectification error of lateral designs is kept low by both maximum damping and a very fast dead-beat controller [18] loop with a bandwidth of well above 2 kHz. The drawback is higher thermomechanical noise.

### 2.3. Production Data

For the evaluation of production data, the following two settings representing two different accelerometer products of NG LITEF were evaluated (see Table 1).

It is important to mention that the following results and the conclusion are specific for the two MEMS accelerometers from NG LITEF. They are calibrated in a temperature range from −40 °C to 85 °C. The parameters scale factor, bias, and misalignment are determined with a static multi position test [19] at various temperatures during heating and cooling. Effectively, a progression of these parameters with temperature is obtained and loaded into the accelerometer’s digital signal processor (DSP) as compensation models. The following residual errors over temperature are measured and verify the compensation in a final acceptance test.

#### 2.3.1. Scalefactor Residual Error

According to the theory presented above, the accelerometer scale factor of lateral designs should have a smoother progression over temperature for two reasons: First, it is independent from the actual pendulum position, and secondly the electrode distance is not sensitive to changes of the outside pressure. Looking at data obtained with the two different designs, one finds an improvement in scale factor residuals over temperature by more than a factor of two for the lateral design with currently 50 ppm in average.

#### 2.3.2. Bias and Bias Residual Error

The raw bias of lateral designs should be higher due to their lower sensitivity to acceleration. Effectively, tiny asymmetries in the MEMS chip or stray capacities in any place lead to a high bias. The comparison of production data from vertical and lateral design indeed reveals a raw absolute bias value that is a factor 3.5 higher for the lateral design with about 300 mg average. Nevertheless, the bias variation over temperature is about 30% smaller for the lateral design which shows a temperature sensitivity of the bias of about 20 µg/K.

This positive result is mainly due to the fact that, during the development of the lateral design for series production, packaging topics and layout issues were improved significantly. Consequently, the bias residual error over temperature was also improved by about 30% to 65 µg on average.

#### 2.3.3. Vibration Rectification Error

The vibration rectification error of NG LITEF’s AHRS product LCR-100 [20] is monitored regularly in production in a vibration performance test. This test allows for comparison of accelerometers with the two different design approaches. As expected, it turns out that the lateral design is superior to the vertical design. This statement is true for all vibration levels. While the improvement in vibration rectification error is only about 20% at highest vibration levels (above 10 g_rms_ at sensor level), the impact of displacement independent forces can be seen even more at the lowest vibration levels where highest performance is required. At these vibration levels, the lateral design outperforms the vertical design by more than a factor of five.

### 2.4. Summary and Outlook

Two different designs of MEMS accelerometers were compared on a theoretical level and by analyzing data collected during series production of both designs at NG LITEF. In summary, the newer design operating with lateral displacement shows an improved performance in comparison to the older design with vertical displacement. Some of the performance improvements were expected per design, such as scale factor and vibration performance at low vibration levels. Others were reached by carefully adapting the overall accelerometer concept to the special weaknesses of lateral designs, such as low damping and lower sensitivity. The impressive results for the lateral design are partly due to the fact that wet etching technology is applied for the vertical design. A comparison with a vertical design, processed with DRIE, would probably come out more balanced.

In the future, two aspects of high performance MEMS accelerometers will be improved:

1. The excellent behavior of the current lateral design in heavy vibration environments will be improved by further increasing the bandwidth of the accelerometer while the force independency of the design will be maintained.
2. The acceleration sensitivity of the MEMS chip shall be increased to improve the bias stability of the system. This can be achieved by either a chip redesign or by electrostatic manipulation of the accelerometer’s spring constant. While the former concept is a more general task to be performed as an iteration of the current MEMS chip design, the latter can be applied already on existing chip designs.

However, we recognize there is a trade-off between the two aspects.

## 3. MEMS Gyroscopes

### 3.1. Technology

**Figure 3:**
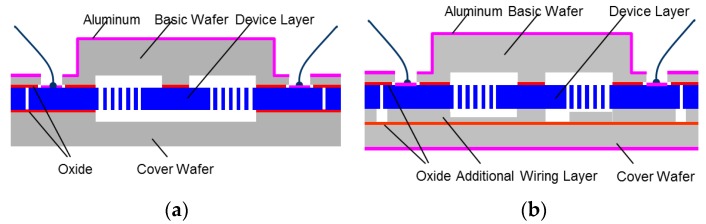
Gyroscope Chip Technology: (**a**) Classical 3-layer SOI; (**b**) Improved Bridge Technology.

The technological processes are identical for our MEMS accelerometers and gyroscopes and are based on DRIE and Silicon Fusion Bonding as already explained in Section 1 and Section 2.1. However, recent major improvements have been achieved by the insertion of an additional routing layer through wafer-bonding of conductive silicon on silicon in addition to silicon on insulating silicon dioxide [21] as depicted in Figure 3. Research groups have reported similar approaches [22]. As a major step forward this, MEMS-bridging technology provides conductive access to inner MEMS electrodes without sacrificing valuable degrees of freedom in micro-mechanical design. Open C-frame shaped Coriolis-sensitive masses can be circumvented and thus the associated parasitic effects of pincer movements.

With the bridging technology, we manage to achieve quality factors above 0.1 Mio. without a getter and measure the quality factor with a precision of better than 0.25% with relative measurements before and after aging stress and monitoring storage. This is accomplished on a series production scale for every single MEMS gyroscope and can thus confirm long-term stability over product lifetime.

### 3.2. Gyroscope Chip Design

**Figure 4:**
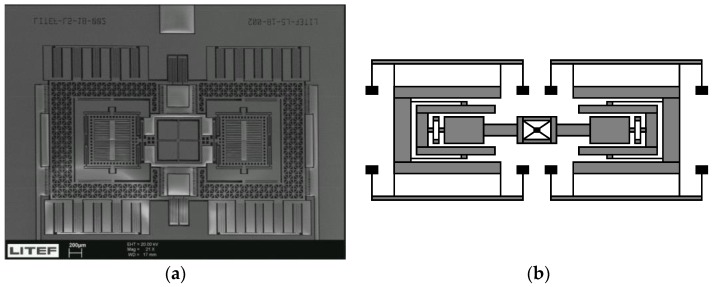
Gyroscope Design: (**a**) SEM image showing the dual mass Lin-Lin-structure with rotational spring; (**b**) Mass-suspension scheme.

For our latest MEMS gyroscope design, the main focus was on robustness with respect to environmental conditions for which MEMS are known to be inherently susceptible. Many MEMS manufacturers have reported excellent results for bias under benign conditions. However, under real operating conditions over temperature, vibration, acoustic noise, cross-axis coupling, and aging, the availability of performant devices quickly narrows. We have addressed the above by a dual-mass gyroscope with dedicated rotational coupling and intentional decoupling of the Coriolis-sensitive parts from the excitation oscillation [23]. We found that by using special linear acceleration control loops operated in the DC-frequency-band for each of the two Coriolis-sensitive masses, only an approximate 8–10 factor of reduction of vibration rectification coefficient could be achieved. While by strong rotational spring coupling in the gyro-chip, a reduction of two orders of magnitude has been achieved. Figure 4 depicts the micromechanical structure (a) and the strongly coupling rotational spring (b).

Acoustic sensitivity is strongly related to vibration sensitivity however, the predominant parts of vibration spectra typically range from 0 Hz to about 1 kHz, while the predominant acoustic spectra range from 0 Hz to about 8 kHz and can therefore directly influence the MEMS-oscillations. The strong rotational spring coupling also reduces the acoustic sensitivity, and a simple measure is to keep the resonant frequency out of the acoustic spectrum. Additionally, a carefully chosen chip mounting on the module assembly level further mitigates acoustic noise susceptibility. For the targeted tactical-grade performance, we found the measures to be by far sufficient, however it is evident that a quad mass design could be superior for higher-precision MEMS gyros as they are not only force balanced, but also torque balanced with respect to an outer frame [24].

Aging effects are very hard to model. With class II gyros such as quad mass gyros, methods for dynamic self-calibration have been proposed [25] and promise a new revolutionary MEMS gyro performance range. However, there are also drawbacks in terms of complexity and redundancy and the alternative is to reserve aging margins in the specification and to monitor key characteristics during the manufacturing process. This is especially true for the enclosed vacuum, as it can be seen from the error model in [26] that the stability of the resonator quality factors directly affects the long-term stability of the instrument bias.

### 3.3. Operating Principles

Various MEMS manufacturers employ Delta-Sigma-modulation schemes to operate MEMS gyroscopes (see e.g., [27,28,29]). Major benefits are low size and low power consumption. The switching typically takes place between only two voltage levels.

A similar pulse modulation scheme is ternary pulse modulation [30,31], which employs three voltage levels. The use of positive, negative, and zero voltage levels provides additional degrees of freedom for pick-off electronics and can completely avoid accumulation of bound charges along oxide planes in less-sophisticated chip structures.

The introduction of electrostatic springs for frequency matching is simple when a separate set of electrodes is provided, or alternatively—as in our case—a time multiplex modulation scheme is employed using D/A-converters and a single A/D converter. We found this tradeoff suitable to achieve very high performance, while sacrificing some size and power consumption. Our highly-optimized time multiplex scheme achieves the following functions with quasi-parallelism: (i) Excitation mode drive; (ii) excitation mode pick-off; (iii) detection mode rate and quadrature rebalancing; (iv) detection mode pick-off; (v) detection mode resonant frequency matching with excitation mode; and (vi) DC quadrature control of detection mode. All voltage stimuli are summed at a single charge amplifier connected to the proof mass’s silicon layer and are demultiplexed and demodulated by signal processing in a digital signal processor or FPGA/ASIC.

Due to MEMS-chip imperfections such as asymmetries, the quadrature over temperature becomes a performance limiting factor. The time multiplexed scheme therefore provides quadrature compensation components in the DC and AC domains, where the DC-regulator is the slower control loop compared to the AC loop by far, with bandwidth still high enough to follow temperature changes.

The major control loops for control of constant excitation oscillation and for force-rebalancing of the detection oscillation are operated in the bandpass domain and are augmented by noise shaping at the torquers. The overall behavior is very much comparable to a ternary pulse or Delta-Sigma-modulation technique, however avoiding auxiliary electrodes for special functions.

As a background task, the excitation resonator frequency is tracked to derive clock and demodulation signals. In addition, artificial out of band noise and a sensor model are used to estimate the basic detection resonator parameters. The local temperature at the MEMS gyro can be monitored very precisely by using the natural resonance frequency sensitivity of the silicon. Research groups have reported similar approaches [32].

### 3.4. Performance and Outlook

**Figure 5:**
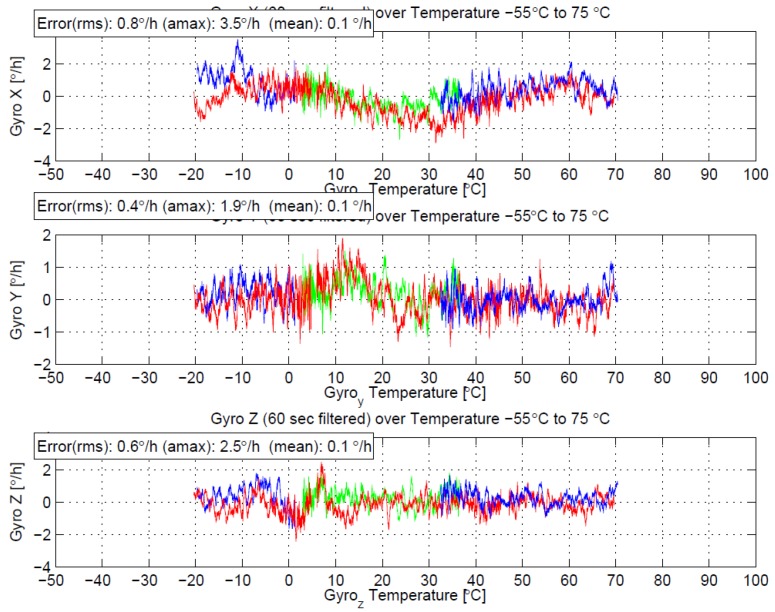
MEMS gyroscopes temperature-cycled and evaluated in our MEMS-IMU SN1880 as a test vehicle and evaluated in the temperature range from −20 °C to +70 °C ambient. Temperature was stabilized at −15, −54, −10, +30, +75, and +10 °C for 2 h, with intermediate ramps of 1 K/min. The three subgraphs show 60 s median filtered rotation rate data in [°/h] over temperature in [°C] for the three gyro axes X, Y, and Z respectively. The three temperatures were measured locally at each gyro. The 1σ bias errors over temperature of the three gyroscopes are 0.8, 0.4, and 0.6°/h respectively.

So far, with our MEMS-IMU product [33] on a series production scale, a performance of 10°/h (1σ) residual bias for the MEMS gyroscopes has been specified in the datasheet. With the optimized technology, design and operating principle described above, MEMS gyroscopes have been assembled into our MEMS-IMU as a research test vehicle. The recent improvements indicate that bias errors over temperature <1°/h are within reach for series production. Example data of three optimized gyroscopes measured in the MEMS-IMU over temperature and evaluated in the range of −20 °C and +75 °C ambient temperature are shown in Figure 5. To our knowledge, such performance for a triad of silicon MEMS gyros in an IMU has not been published before.

With further optimizations through improvements in algorithms and auxiliary control loops, we expect that 0.5°/h MEMS dual-mass gyroscopes should be feasible on a broad series production scale. The next true MEMS revolution with a jump in performance over about two orders of magnitude however, is likely to rely on class II quad mass gyroscope designs [25].

## 4. Fiber Optic Gyros

### 4.1. Background

For more than 25 years, NG LITEF has been continuously improving its Fiber Optic Gyroscope products based on its breakthrough closed loop signal processing technology with random modulation and auxiliary control loops [13], the Multifunction Integrated Optics Chip (MIOC or I/O Chip) and dedicated coil winding and potting technology. Two mature FOG architectures have evolved: (i) the 0.05°/h class of FOG triads with cooled superluminescent diode (SLD) light source used in the NG LITEF AHRS, marine and land navigation products [20]; and (ii) the 1°/h to 6°/h class of single axis µFORS FOGs suitable e.g., to build dedicated IMUs and for stabilization applications operating under harsh environmental conditions.

The MIOC chip is fabricated in compliance with quality standard DIN/ISO 9001 in NG LITEF production laboratories using Lithium Niobate (LiNbO_3_) as the material basis. Since the early 1990s more than 190,000 different types of optical chips have been produced for our own use in Fiber Optic Gyroscopes and for systems based on our FOGs. The MIOCs are manufactured using the annealed proton-exchange technique on x-cut Lithium Niobate wafers [34] and integrate the standard functions of (i) a polarizer; (ii) a main coupler/beam splitter; and (iii) a broadband electro-optical push-pull phase modulator on a single chip.

**Figure 6:**
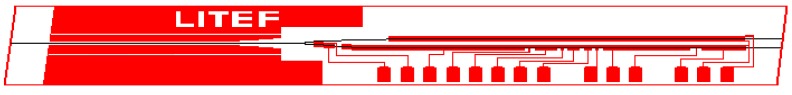
Waveguide and Electrode structure of a mixed signal 12-bit MIOC.

For miniaturized single axis Fiber Optic Gyroscopes of the µFORS product family, modified MIOCs were developed to further optimize integration efficiency. The so-called Mixed-Signal MIOC [35] which integrates, in addition to the standard functions mentioned above, a full 12-bit Digital-to-Analog Converter. The latter is realized through 12 binary-weight divided electrodes for electro-optical phase modulation and an additional small electro-optical phase shifter (see Figure 6). The advantage of the binary-weight divided electrode structure of the phase modulator is the possibility to drive the electrodes from our digital ASIC [11] with no further need of an additional DAC.

In the digital ASIC, look-up RAM is implemented which can be used to correct bit errors of all individual digital electrodes to maximize the accuracy of the optical Digital-to-Analog Converter [36]. By introducing this feature, not only can all bit errors be minimized but also the use of non-binary or the combination of binary/non-binary electrodes would be possible. The combination of binary/non-binary electrodes also allows 16-bit resolution with at least 15-bit accuracy for the optical D/A converter without increasing the chip length and cost. Additionally, the look-up table can be used to optimize the linearity of the phase modulation.

**Figure 7:**
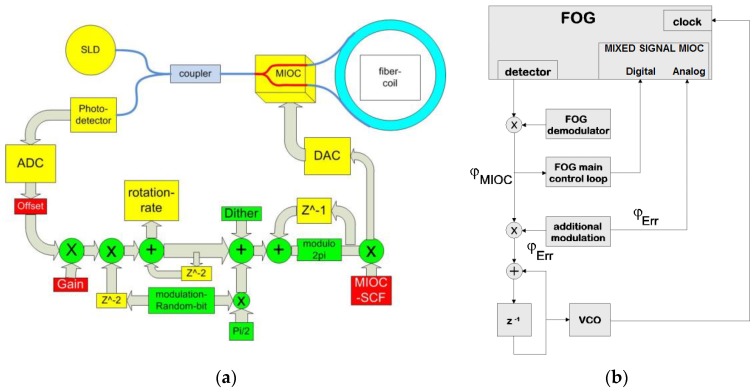
(**a**) Block diagram showing a single-axis closed-loop FOG including signal processing datapath; (**b**) Block diagram showing FOG modulation frequency tracking.

The additional small phase shifter of the Mixed-Signal MIOC introduces an optical phase shift of π/32 which is used as an input for the control of the modulation frequency tracking [37]. Most FOG properties depend on temperature and tolerances always exist in the production process of the fiber coil. However, if the modulation frequency of a FOG is maintained at a fixed constant cycle time, that cycle time generally does not match the momentary transit frequency of the light passing the FOGs optical path, making the FOG susceptible to synchronous crosstalk that contributes to the bias error. To adapt the modulation frequency to the actual conditions over temperature, the transit time of the light can be used to obtain a way to control the modulation frequency. For that reason, the additional modulation correlated to the detected signal allows the operation of an auxiliary control loop for a Voltage Controlled Oscillator (VCO) which for its part is used to control the internal modulation frequency. The concept is shown in Figure 7 and technically realized by a Direct Digital Synthesizer (DDS).

On average, a three-fold bias improvement on a series production scale has been achieved for our µFORS products through this method and experiments showed that which such improved Mixed Signal-MIOCs the performance of navigation grade Fiber Optic Gyroscopes can be achieved.

**Figure 8:**
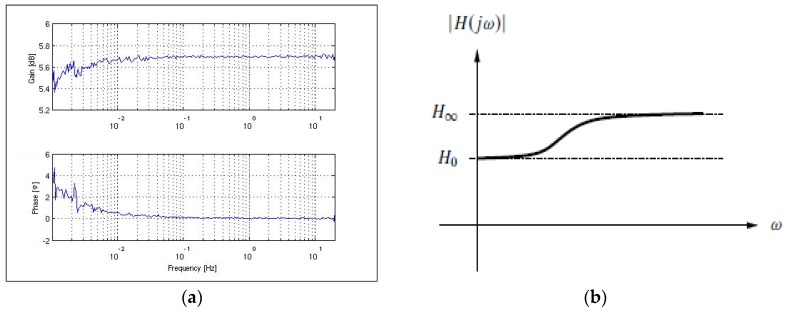
(**a**) Example of a measured electro-optical transfer function of LiNbO3 modulator chip with logarithmic scaling of frequency axis; (**b**) Derived model of the high-pass-type transfer function of a non-ideal phase modulator with linear scaling of the frequency axis.

Lithium Niobate and modified Lithium Niobate with annealed proton exchange waveguide formation show a low intrinsic conductivity which results in a high-pass-type behavior of an electro-optical modulator also termed phase bleed. The typical cut-off frequency is lower than 1 mHz (−3 dB value) as shown in Figure 8. The high-pass-type behavior of the modulator chips has, so far, limited the achievable angle random walk coefficient with closed loop operation in very sensitive applications. Only recently, NG LITEF developed a DC-free modulation scheme (see the following sections) to avoid low frequencies in the random modulation signal. A special new MIOC supports this modulation scheme with a relatively small push-pull half-wave voltage of 1.7 V on a moderate chip length of 30 mm.

Furthermore, the design of all MIOCs produced at NG LITEF is optimized to avoid residual intensity modulation coming from the interference of a secondary light path in the chip with the light paths in the modulated waveguides. Additionally, on all MIOCs the Z-faces of the crystal are coated with a conductive layer to short out the pyroelectric effect and to achieve high stability over strong temperature ramps [38].

### 4.2. Random Modulation and MIOCs with Phase Bleed in the FOG

The technical concept of random modulation for the FOG is discussed in detail in reference [39] and its basics in [40,41,42,43]. The problem of the insensitivity sectors (also called dead zones) of the fiber optic gyroscope is brought up in [39,44,45] and at the same time the solution to the problem applying random modulation is stated. This solution is due to an improvement to the modulation procedure, which is controlled by a random data generator and which is applied to the correlation freedom of the demodulator reference signal.

**Figure 9:**
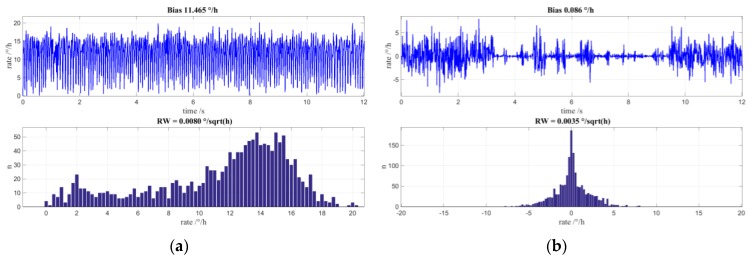
(**a**) Measured time signal and value distribution histogram for low applied rotation rate of approximately 12°/h; (**b**) Measured time signal and value distribution histogram for special case of applied rotation rate of 0°/h.

It was from the results of constructed Fiber Optic Gyroscopes that the effects of MIOC phase bleed in a closed loop FOG with random modulation was first detected. In an ideal gyro experiencing a constant applied rate, the digital output would be centred on the applied rate with values symmetrically above and below this value. However, the situation for low input rates did not have a normal distribution but exhibited a preference for values in the region of Ω = 0. This is illustrated in Figure 9 in which each top graph shows the gyro output over time and the lower graph is a distribution of the reported values.

In the graph of Figure 9a, the input rate of the gyro is located at approximately 12°/h. Clearly, this is not a normal distribution about the applied rate and is rather unbalanced with values that extend into Ω = 0. If the input rate is set to zero then, as is shown in Figure 9b, there is no normal distribution in the measured values but, again, an emphasis of measurement data around Ω = 0. The time plot clearly shows regular constrictions of the rotational speed from which the measurement distribution arises. NG LITEF circumvented this behavior by introduction of an additional dithering modulation that also drives the FOGs auxiliary gain control loop, however at the cost of a basic limitation of achievable angle random walk that persisted until recently.

As explained in Section 4.1, the real behavior of the phase modulator can be modelled with high-pass characteristic as shown in Figure 8b and implemented into our FOG sensor simulation model. For a frequency ω = 0—and also at very low frequencies—the phase modulators have a lower gain than at higher frequencies.

**Figure 10:**
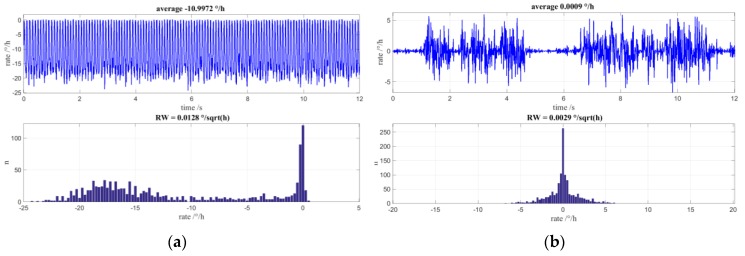
Simulations including non-ideal MIOC transfer function; the dithering for auxiliary gain control loop is off to emphasize the effects of MIOC phase bleed: (**a**) Simulated time signal and value distribution histogram for a low applied rotation rate of approximately −10°/h; (**b**) Simulated time signal and value distribution histogram for applied rotation rate of 0°/h.

Figure 10a shows simulated rate values and their distribution for an applied rate of −10°/h. The trend of the output rate towards 0 is clearly visible. In Figure 10b are the rate values and the distribution for a zero-input rate. Here, too, the preference of the initial rotation rates is zero and it is apparent that the rate value signal is constricted.

### 4.3. Approach with DC-Free Modulation

#### 4.3.1. Preliminary Considerations

**Figure 11:**
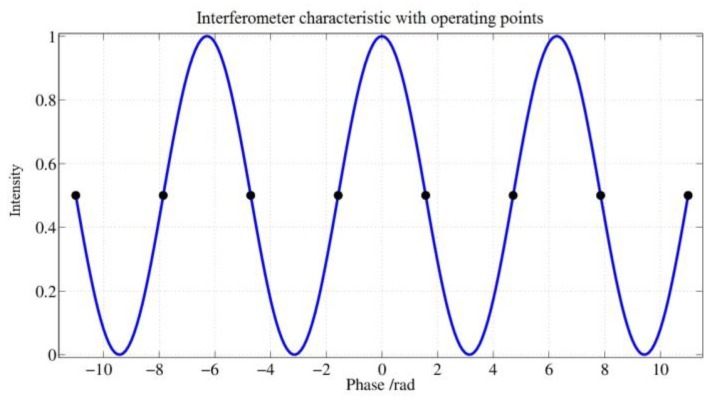
Sagnac Interferometer characteristic with operating points showing the relative intensity over the Sagnac phase in radiants.

Figure 11 shows the intensity over phase characteristic of the Sagnac interferometer.

During the operation of the fiber optic gyroscope, the marked operating points are always controlled by the modulation signal at the MIOC. Regarding the choice of the sequence of the operating points, there are certain degrees of freedom that require careful consideration. These are expressed through the following items:

1. Without modulation, the peak of the interferometer characteristic would be steered where the slope is zero. Thus, the sensitivity of the sensor is also zero and there would be no directional information present. To avoid these disadvantages, the control uses points that lie where the slope is greatest.
2. If only points with the same sign were controlled, an applied rotation rate would lead to a DC voltage signal at the photodetector, which would be suppressed by the subsequent circuit stages. Therefore, work points with changing signs are controlled. This results in a modulation of the detector signal so that the signal lies in the transmission range of the following amplifier stages.
3. If the control of the working points alternates periodically between positive and negative slopes, the drive signal at the MIOC would correlate with the demodulator reference of the detector signal. This results in an insensitivity in the sensor for small rotation rates. Therefore, the sequence of the signs of the slope at the operating points have to be chosen in a way that the mentioned correlation disappears.
4. The modulation must be such that a stable operation of the scale factor controller (auxiliary control loop) can be ensured for any input rotation rates of the sensor.

The MIOC has a lower transmission factor at very low frequencies than at medium and high frequencies. If the low frequencies and DC voltage were kept away from the MIOC, then the sensor errors must disappear since the drop of the transmission factor at low frequencies can no longer have an effect.

The concept, therefore, is to modify the modulation scheme or, better, the statistical properties of the controlled work points so that the modulation signal at the MIOC has the mean eliminated; that is: it is a mean-free modulation as the expectation value would be zero. In addition to the above four objectives, a fifth item arises:

5. The modulation must be such that the expected value of the MIOC drive signal becomes zero.

However, it is now apparent that in the above-described prior art method, the degrees of freedom have been exhausted by specifying the first four objectives. Therefore, should the fifth property be applied, this would be at the cost of the first four objectives. In particular, it is expected that the freedom from correlation between the MIOC control signal and the demodulator reference would disappear. This is a prerequisite for the disappearance of the lock-in effect during over-coupling from the MIOC signal to the detector signal. This has been confirmed by simulation. This statement applies to the method in the current technical concept with a modulation range of the MIOC signal of 2π. However, it is now possible to increase the control range of the MIOC signal to 4π and thereby increase the number of degrees of freedom which can be used in controlling the work points. In this case, all five of the above-mentioned objectives can be achieved. The resulting solutions for the 4π control range of the MIOC are presented in the following sections.

#### 4.3.2. Statistical Rounding

**Figure 12:**
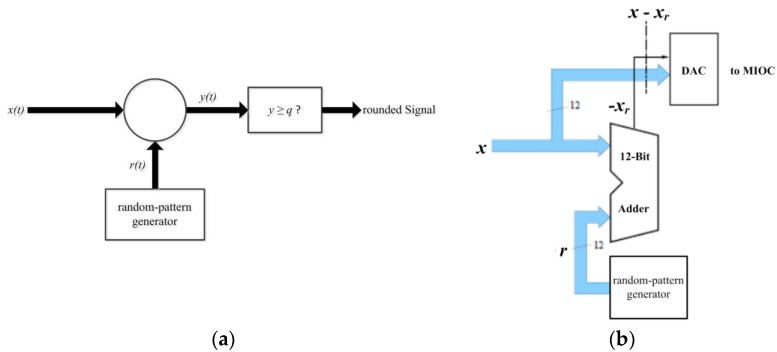
(**a**) Principle of statistical rounding; (**b**) Resulting optimized circuit realization for driving the MIOC.

**Figure 13:**
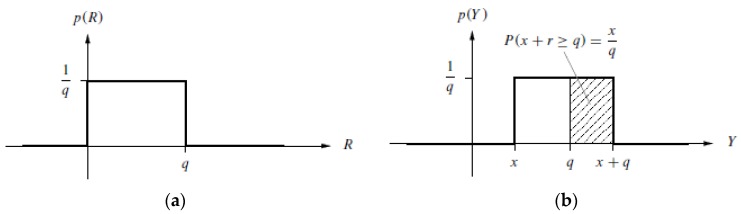
(**a**) Probability distribution function of the random variable *R* (random numbers *r*); (**b**) Resulting distribution function of random variable *Y* (random numbers *y = x + r*).

The mean value freedom of the MIOC control signal can be achieved by so-called statistical rounding [46]. As shown in Figure 12a, the wanted signal x∈[0, q) is mixed with a random number with a distribution shown in Figure 13a.

A comparator then checks whether the output signal meets the y ≥ q condition. The probability P(y ≥ q) of the hatched area of Figure 13b is

(6) $$P\left(y\;\geq \;q\right)\;=\;\frac{x}{q}\;.$$

This constructs the rounded signal

(7) $$x_{r}\;=\;\{\begin{array}{c} 0\;\mathrm{for}\;y\;<\;q\\ q\;\mathrm{for}\;y\;\geq \;q \end{array}\;.$$

Thus, the expected value for $x_{r}$ is

(8) $$E\left(x_{r}\right)\;=\;P\left(y\;<\;q\right)\cdot 0\;+\;P\left(y\;\geq \;q\right)\cdot q\;=\;\left(1\;-\;\frac{x}{q}\right)\cdot 0\;+\;\frac{x}{q}\cdot q\;=\;x\;.$$

The rounded signal $x_{r}$ thus corresponds to the input signal x. If signal ${x\;-\;x}_{r}$ is formed, then its expected value is

(9) $$E\left({x\;-\;x}_{r}\right)\;=\;0$$

As a basic concept, q = π or q = 2π is selected and the MIOC, instead of a signal x, is fed with a mean-value-free signal, ${x\;-\;x}_{r}$. For q = π, the transition from x to ${x\;-\;x}_{r}$ changes the MIOC signal. Hence the phase of the interferometer is changed by the value π, which changes the operating point to an operating point with an opposite slope. This measure changes the demodulator reference. For the situation of q = 2π, there is also a change of the operating point but the slope is unchanged. Owing to the periodicity of the interferometer characteristic, there are no other changes. Figure 12b shows the circuit realization of statistical rounding for mean-free-value $\left(E\left({x\;-\;x}_{r}\right)\;=\;0\right)$ of the MIOC signal.

The unmodified signal is x(t) ∈ [0,q). This, for example, could have a word width of 12 bits. The weighting of the MSB is $\frac{q}{2}$. To this signal, x(t), is added an equally-distributed random number which also has the same word width of 12 bits. The condition x + r ≥ q is signaled by the carry bit—the sum itself is not used. If the signal is now formed into a 13-bit number, with the carry bit forming the new MSB, then this number can be interpreted as a two’s-complement number and is mean-free since the new MSB has the weight of –q. The signal ${x\;-\;x}_{r}$ with $E\left({x\;-\;x}_{r}\right)\;=\;0$ can be now fed to a D/A-converter which, in turn, controls the MIOC. As already explained above, two different cases are possible:

1. q = π which results in a modulation range of the MIOC of 2π (henceforth referred to as 2π-modulation).
2. q = 2π which results in a modulation range of the MIOC of 4π (henceforth referred to as 4π-modulation).

**Figure 14:**
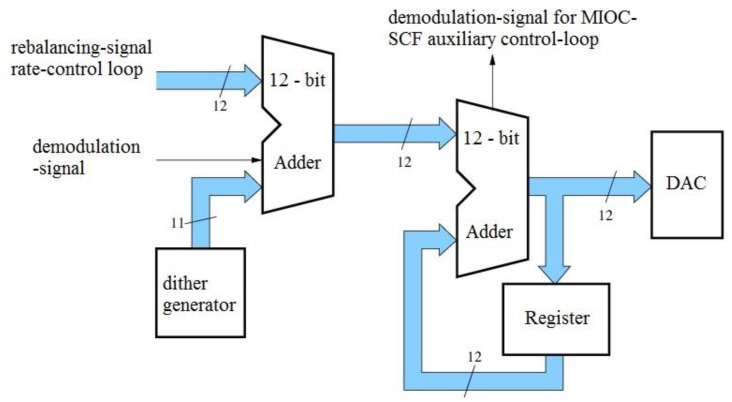
Modulation stage and phase accumulator used in the current design.

It will now be shown how these modifications are incorporated into the current technical concept. This related part of the circuit is shown again in Figure 14.

#### 4.3.3. Mean-Value-Free MIOC Control with 4π-Modulation

**Figure 15:**
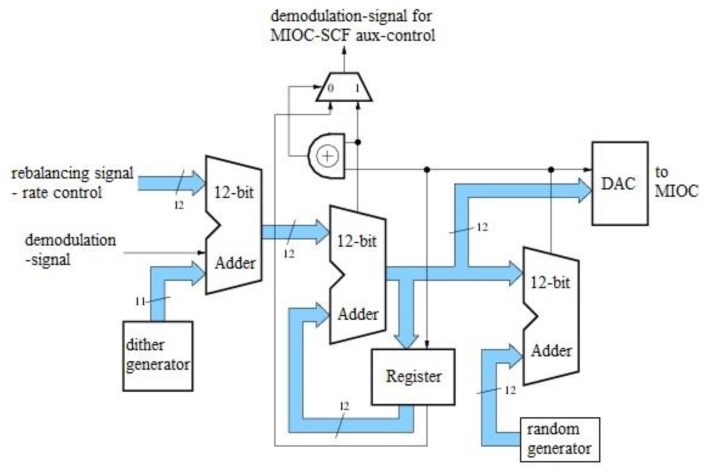
Configuration for mean-value-free 4π-modulation.

As already mentioned, compromises would be made in the implementation of mean-value-free 2π-modulation since the number of degrees of freedom was not sufficient for the control work points. This goal—a mean-value-free control of the MIOC—can be achieved by surrendering other conditions. If a control range of 4π for the MIOC signal is considered, then all simultaneous conditions for a correlation-free demodulation signal and a mean-free MIOC signal can be fulfilled. For statistical rounding, a quantization of q = 2π is chosen. The entire data path remains unchanged. Only here the D/A converter receives an additional bit as MSB with the weighting of 2π. This range expansion achieves a mean-value freedom at the D/A converter. The additional bit shifts the controlled operating points by an amount of 2π, which does not change the sign of the slope of the respective operating point, and thus leaves the demodulation signal unchanged. Thus, the non-correlation of the demodulation signal is restored but the MIOC control is now mean-value-free. However, the generation of the demodulation signal for the scale factor controller has to now be adapted for the new method. Figure 15 shows the resulting configuration.

**Figure 16:**
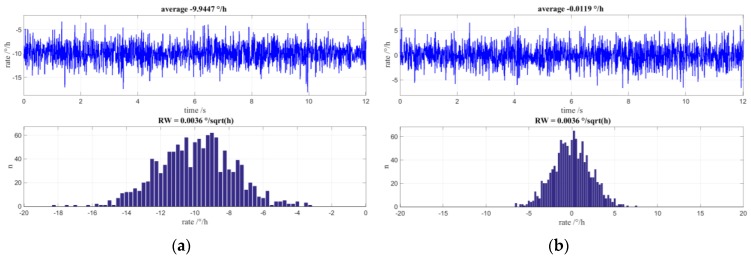
Simulations including non-ideal MIOC transfer function and mean-value-free 4π-Modulation: (a) Simulated time signal and value distribution histogram for an applied rotation rate of approximately −10°/h; (b) Simulated time signal and value distribution histogram for applied rotation rate of 0°/h.

**Figure 17:**
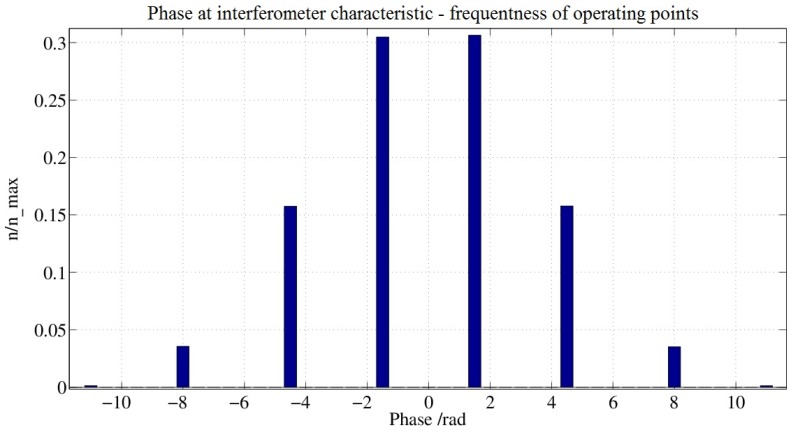
Frequency distribution of operating points with mean-value-free 4π-modulation.

The implementation is slightly simplified since there is now no longer any effect on the demodulation signal (this is stated again for emphasis). Note that the signal at the output of the phase accumulator is subjected to statistical rounding with q = 2π and that the additionally-obtained bit is fed as an MSB to the one-bit-wider D/A converter. In this example, the additional bit is also fed back into the register of the phase accumulator. This is necessary to derive the demodulation signal for the scale factor controller. Figure 16a shows the rate and value distribution at applied rate of −10°/h. Similarly, the rates and value distribution for an input rate of zero are shown in Figure 16b. Both figures show a normal distribution of values. Figure 17 shows the frequency distribution of the controlled operating points. The number of operating points has doubled from four to eight.

### 4.4. Discussion and Outlook

Our method demonstrates that FOG sensor errors in the form of periodic phases can be fed back with a sensor output signal from zero to a frequency-dependent transmission function of the MIOC with attenuation at low frequencies. These errors can be eliminated, if low frequencies and DC voltage are kept away from the MIOC by a mean-free drive.

It has also been shown that the mean-value freedom of the MIOC control signal can be produced by subtracting a signal quantized by q = π or q = 2π, obtained by statistical rounding of the original MIOC control signal. When q = π, this results in a MIOC control range of 2π. Mean-value-free range of freedom can be produced here only by modifying the modulation scheme, whereby other sensor properties can be impaired. Setting q = 2π gives a MIOC control range of 4π. In this case, the mean-value-free state can be achieved without detriment to the modulation scheme and thus without adversely affecting other sensor properties.

Breaking the MIOC phase-bleed-induced noise limitation through means of an improved and efficient signal processing has paved the way for further FOG applications with navigation grade demands.

## 5. Comparison of Fiber Optic and MEMS Gyroscopes

Both types of sensors are equally suitable to build strapdown navigation and AHRS systems. Since MEMS gyroscopes have entered the high-end tactical grade performance of 1–5°/h they have started to replace some Fiber Optic Gyroscopes due to their inherent cost, size, weight, and power (CSWaP) advantages. Nevertheless, there are a number of implications that need to be considered during the selection process.

From the fact that Fiber Optic Gyros do not contain moving parts it can be concluded that they perform generally much better over vibration, shock, and acoustic noise [µFORS]. Nevertheless, there is a sensitivity of the fiber coil towards mechanical stress that can be considered a temporary stress-induced Shupe effect [47]. However, this sensitivity only becomes apparent for performance ranges where MEMS gyroscopes are not available yet.

Temperature related Shupe effect and magnetic sensitivity due to the Faraday effect are disadvantages of the FOG and advantages of the MEMS gyroscope. These types of environmental conditions can be shielded from FOG coils, however the mechanical construction and calibration effort generally affect size and cost.

FOG noise is widely affected by the light source, electronics, and the signal processing scheme, while MEMS gyroscope noise is widely affected by the moveable mass and also electronics and the signal processing scheme. While MEMS are inherently associated with small size, it is unlikely that they will outperform FOG noise performance on a large scale. Nevertheless, MEMS performance can be scaled with size.

One of the greatest advantages of FOG gyroscopes over MEMS gyroscopes is the achievable bandwidth. We have accomplished several hundreds of Hz bandwidth with MEMS, however FOGs due to their interferometric nature and simple deadbeat control scheme offer merely infinite bandwidth, only limited by the interface sampling frequency. For applications with very fast control loops—e.g., in some military domains—FOG is likely to remain the best choice. The following Table 2 and Table 3 summarize technical parameters achieved within Northrop Grumman LITEF’s MEMS and Fiber Optic Gyroscopes in serial production. After all, it is clear that FOGs are performance-scalable and will continue to remain the predominant gyro sensor for several years in applications that require better than 0.1°/h bias and less than 0.05°/rt(h) angle random walk. Very high performance FOGs can be found in marine systems with large scale factor (~1 km of fiber length and large diameter), where benign environmental conditions can be assumed. Alternatives such as Hemispherical Resonator Gyros (HRGs) and axisymmetric Class II MEMS Gyroscopes are however either already entering the market or are under intensive development.

FOG and MEMS are both mature technologies that are suitable to meet the highest safety standards for aircraft certification. They both provide scalability and are therefore suitable technologies to span over orders of magnitude in performance.

## 6. Conclusions

Based on applications, customer needs, and our long-term experience in the field we revisited the requirements for tactical and navigation grade inertial sensors in navigation applications and presented NG LITEF’s recently accomplished improvements in the domains of MEMS accelerometers, MEMS gyroscopes, and Fiber Optic Gyroscopes to fulfill these requirements. For MEMS accelerometers, lateral structures provide excellent linearity and pressure insensitivity. For MEMS gyroscopes, an optimized chip design and operating scheme provide better than 1°/h residual bias over temperature with excellent vibration and acoustic noise immunity. For Fiber Optic Gyroscopes, the MIOC phase-bleed-induced noise has been eliminated by means of signal processing and the bias over temperature has been improved by continuous adaption to the fiber length. We have also given a comparison of FOG and MEMS gyro technologies to assist system engineers with the process of inertial sensor type selection.

## Figures and Tables

**Table 1:** Details of two different accelerometer products of NG LITEF with different design concepts.

Accelerometer Design	Vertical	Lateral
Structuring	KOH—Wet etching	DRIE
Controller	PI	Dead-beat
Bandwidth	>400 Hz	>2 kHz
Damping	Torquer/Pickoff electrodes	Damping electrodes
Gas pressure	13 mbar	200 mbar

**Table 2:** Typical values achieved by single-axis MEMS gyroscopes for the use in the Northrop Grumman LITEF MEMS IMU. All parameters tested on the IMU level.

Parameter	Test Conditions	Typ.	Unit
Dynamic range		1500 (max.)	°/s
Scalefactor repeatability	−40 °C ≤ T ≤ 85 °C; 1σ	300	ppm
Bias repeatability	−40 °C ≤ T ≤ 85 °C; 1σ	1.2	°/h
Bias instability (Allan deviation)		0.1	°/h
Angle random walk		0.1	°/√h
Vibration rectification error	rms	0.09	°/h/g^2^
−3 dB bandwidth		240	Hz

**Table 3:** Typical values achieved by single-axis FOGs for the use in the Northrop Grumman LITEFs FOG-IMU based systems. All parameters tested on the IMU level.

Parameter	Test Conditions	Typ.	Unit
Dynamic range		1500 (max.)	°/s
Scalefactor repeatability	−40 °C ≤ T ≤ 71 °C; 1σ	30	ppm
Bias repeatability	−40 °C ≤ T ≤ 71 °C; 1σ	0.015	°/h
Bias instability (Allan deviation)		0.0012	°/h
Angle random walk		0.005	°/√h
−3 dB bandwidth		8000	Hz
